# Clinical outcome after open-wedge high tibial osteotomy: comparison of double-triangle locking compression plate (DT-LCP) and T-shaped locking compression plate (T-LCP)

**DOI:** 10.1186/s12891-024-07658-w

**Published:** 2024-08-01

**Authors:** Pengzhao Chen, Jiahao Yu, Haichuan Guo, Peizhi Yuwen, Yanbin Zhu, Yingze Zhang

**Affiliations:** 1 Department of Orthopaedic Surgery, Third Hospital of Hebei Medical University, NO.139 Ziqiang Road, Shijiazhuang 050051, Hebei People’s Republic of China; 2 Key Laboratory of Biomechanics of Hebei Province, Shijiazhuang 050051, Hebei People’s Republic of China; 3 NHC Key Laboratory of Intelligent Orthopaedic Equipment, Shijiazhuang 050051, Hebei People’s Republic of China; 4 Chinese Academy of Engineering, Beijing 100088, People’s Republic of China

**Keywords:** Osteosynthesis, High tibial osteotomy, Minimally invasive surgery, Internal plate fixator

## Abstract

**Background:**

The objective of this study was to compare the clinical outcomes of two internal fixation methods for high tibial osteotomy (HTO): double-triangle locking compression plate (DT-LCP) and T-shaped locking compression plate (T-LCP).

**Methods:**

202 adult patients in our hospital between January 2018 and December 2021 were included and followed up for at least one year: group 1(DT-LCP, 98 patients) and group 2 (T-LCP, 104 patients). Detailed information on demographics, preoperative and postoperative follow-up, surgical procedures, and complications were collected. The information of the International Knee Documentation Committee Knee Evaluation Form (IKDC), Knee Injury and Osteoarthritis Outcome Score (KOOS), Western Ontario and McMaster Universities Osteoarthritis Index (WOMAC) were collected before surgery and at the last follow-up.

**Results:**

A total of 202 patients were included in the per-protocol analysis. No significant difference was found in terms of demographic data between groups, except for age and BMI. Clinically relevant improvements in knee pain were reached up to last follow-up after the operation in both groups. The mean pain scores (KOOS, WOMAC) at the final follow-up were significantly higher among group 1 compared to group 2 (*P* = 0.040 and *P* = 0.023). Furthermore, the DT-LCP internal fixation exerted more excellent effects on other symptoms, function and quality of life than T-LCP internal fixation.

**Conclusions:**

Our study demonstrated that DT-LCP provided better clinical performance due to its implant irritant pain, compared with T-LCP. Thus, DT-LCP is a feasible alternative for the fixation of OW-HTO.

## Introduction

Knee osteoarthritis (KOA) is a common joint degenerative disease and a leading cause of pain and disability in middle-aged and elderly people [1]. Open-wedge high tibial osteotomy (OW-HTO) can provide pain relief and delay the need for total knee arthroplasty (TKA) in young, active, medial compartment osteoarthritis individuals with varus deformity [2–4] and the main advantage of OW-HTO is to avoid common peroneal nerve injury with an overall lower complication rate and the correction size is easy to adjust [5, 6]. However, a stable fixation device is urgent to stabilize the open wedge osteotomy and further to improve bone healing for the instability of the proximal tibia of OW-HTO [7, 8].

**Fig. 1:**
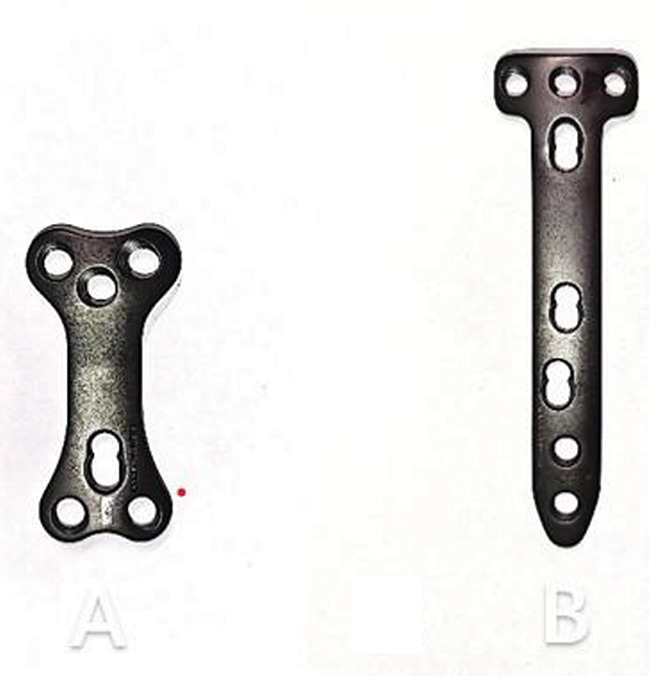
**A**, Double triangle locking compression plate (DT-LCP). The plate is made of titanium alloy. Absolute plate length is 60 mm. **B**, T-shaped locking compression plate (T-LCP). The plate is made of titanium alloy. Absolute plate length is 120–150 mm. In its proximal third, the plate has a T shape

Up to now, there are different plate types for the stabilization of OW-HTO: locking or non-locking plates; short plates or long plates [4, 9–11]. Open wedge osteotomy with long internal fixation has a lower incidence of complications as the stress is more dispersed. Therefore, most surgeons tend to use long-locking plants for the internal fixation of OW-HTO [12–14]. However, the protruding size of the longer internal fixation may lead to discomfort in the early stage of rehabilitation in smaller or thinner patients. Niemeier et al. found that 40% of patients experienced plate-related pain after the use of long internal fixations in HTO [15]. In order to overcome the problem, our team developed a novel and shorter fixation device (a new type of double triangle locking compression plate, Fig. 1A). The double-triangle locking compression plate is designed according to the principle that the triangle has stability. The plate is 60 mm long, 2.8 mm thick, 25 mm wide at the ends and 15 mm wide in the middle. The plate has six screw holes, including five locking holes and one locking compression hole and accepts 5.0 mm titanium locking screws and 4.5 mm titanium cortex screws. The plate is designed to conform to the physiological curvature of the osteotomy site.

At present, there is a lack of clinical outcomes of this new internal fixation system compared with T-shaped locking compression plate (Fig. 1B). Small internal fixation has led to a minimally invasive surgical approach, and the impact of minimally invasive on rehabilitation needs to be evaluated. Therefore, in this study, we investigated the efficacy and complication rate of double-triangle locking compression plate (DT-LCP) with T-shaped locking compression plate (T-LCP) as the control group.

## Materials and methods

### Study subjects

This was a retrospective cohort study from a single center (The Third Hospital of Hebei Medical University) of patients presenting for HTO between January 2018 and December 2021. Patient data were extracted from an existing database. A total of 225 patients were identified in the study period. Patients included in the study completed questionnaires at preoperative and at least one-year postoperative reviews. A total of 202 patients were followed up in this study, and divided into two groups based on internal fixation methods (group 1, 98 patients obtained DT-LCP fixation; group 2, 104 patients obtained T-LCP fixation).

Inclusion criteria were HTO performed for isolated compartment medial OA with varus malalignment, predominantly medial knee pain, age over 30 years. HTO was performed either with DT-LCP or T-LCP, and all patients had a minimum 1-year follow-up.

**Fig. 2:**
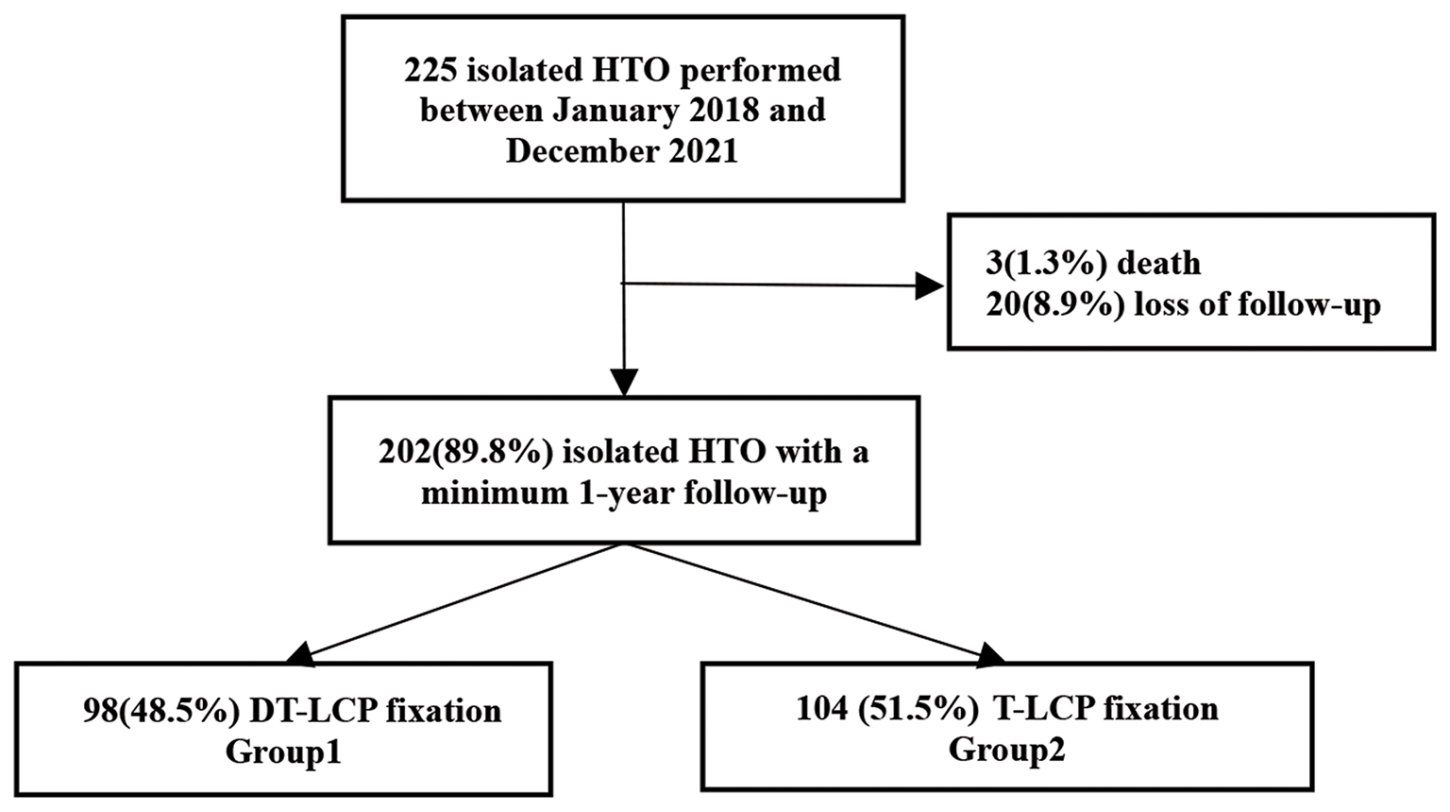
Flow-chart of the study

Exclusion criteria were: HTO performed for non-knee osteoarthritis, renovation of a previous HTO, other additional procedures, such as femoral osteotomy or ligament reconstruction, rheumatoid arthritis, severe osteoarthritis in lateral compartment or patellofemoral compartment, grade 3 collateral ligament laxity, the range of motion of the knee joint is less than 100 °, the flexion contracture greater than 10°, experience of proximal tibial fracture or other surgery, and a previous history of knee joint infection. There are no limitations in terms of radiographic patellofemoral osteoarthritis or body mass index (BMI). A flowchart representing the process of patient selection is illustrated in Fig. 2.

### Surgical techniques

The correction angle was calculated by the Miniaci method [16] and the target mechanical femoro-tibial angle were between 180° and 183° [17]. All cases were examined by intraoperative fluoroscopy to verify the correction effect. Infra-tubercle uniplanar osteotomy was fashioned in the DT-LCP group and biplanar osteotomy technique was followed in the T-LCP group.

### Group1 (DT-LCP)

**Fig. 3:**
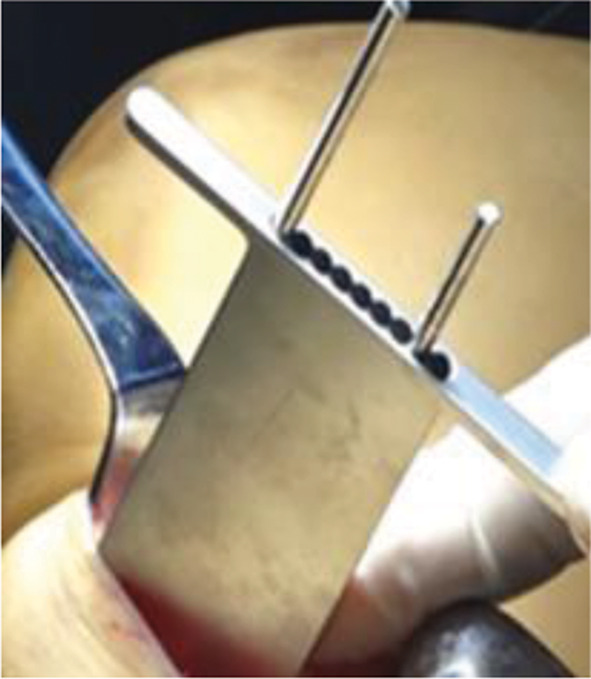
Guide, the instruments used in group1

**Fig. 4:**
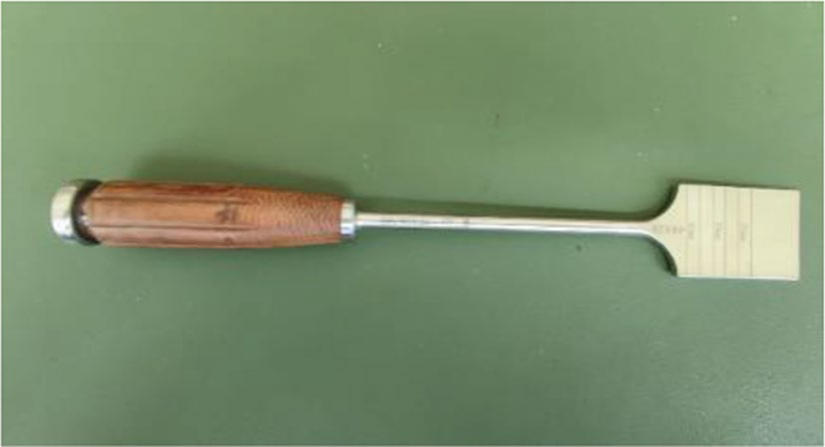
Bone chisel, the instruments used in group1

**Fig. 5:**
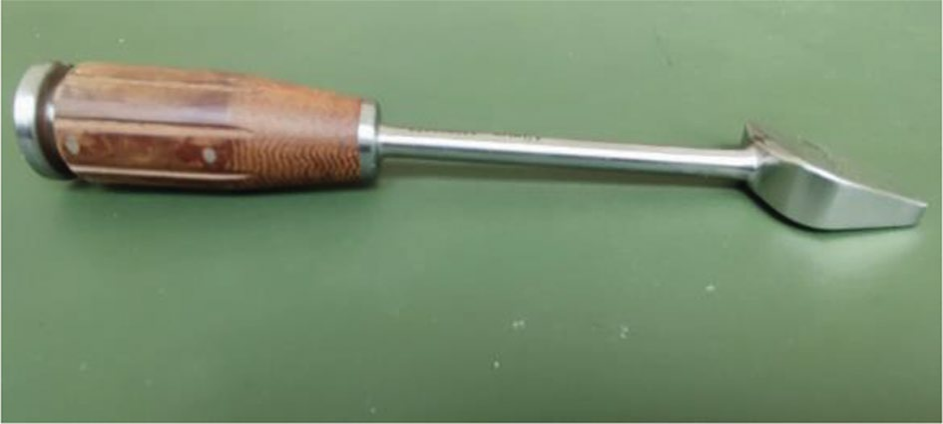
Test mold, the instruments used in group1

**Fig. 6:**
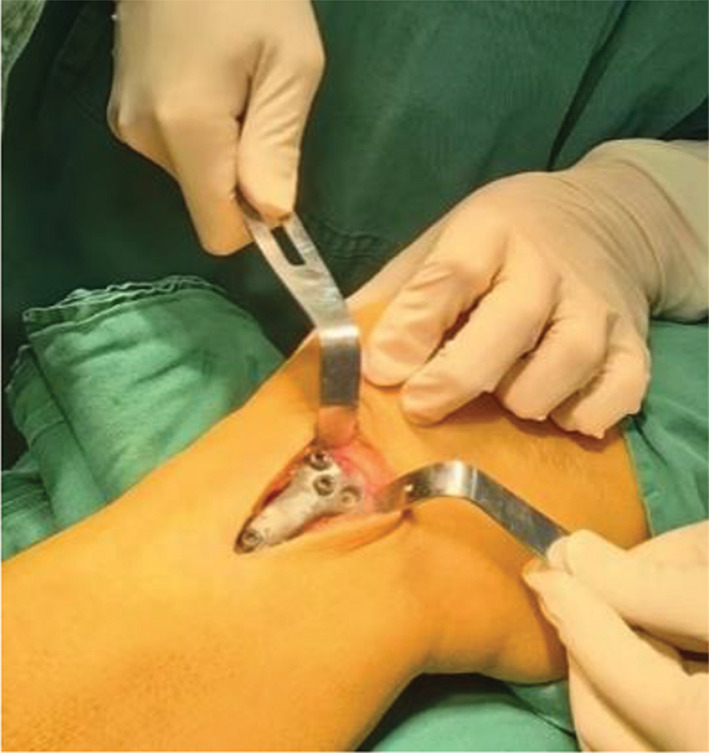
Intraoperative placement of the double-triangle locking compression plate

**Fig. 7:**
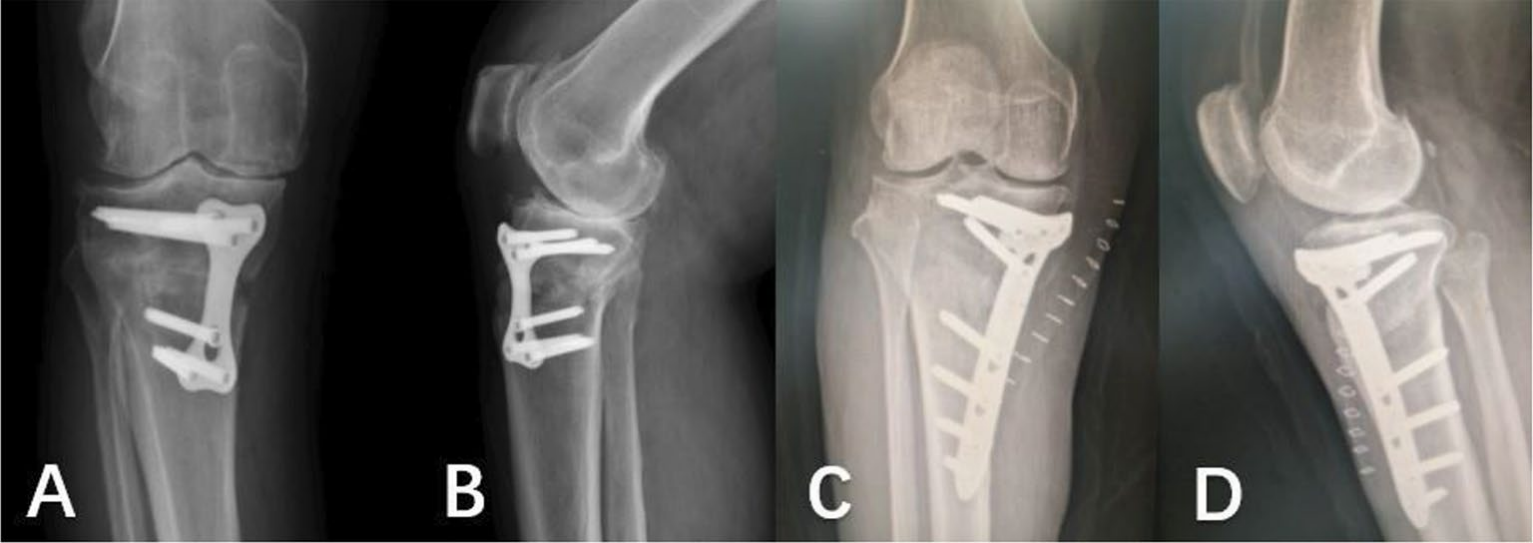
**A**, **B**: DT-LCP frontal and lateral X-rays; **C**, **D**: T-LCP frontal and lateral X-rays

**Fig. 8:**
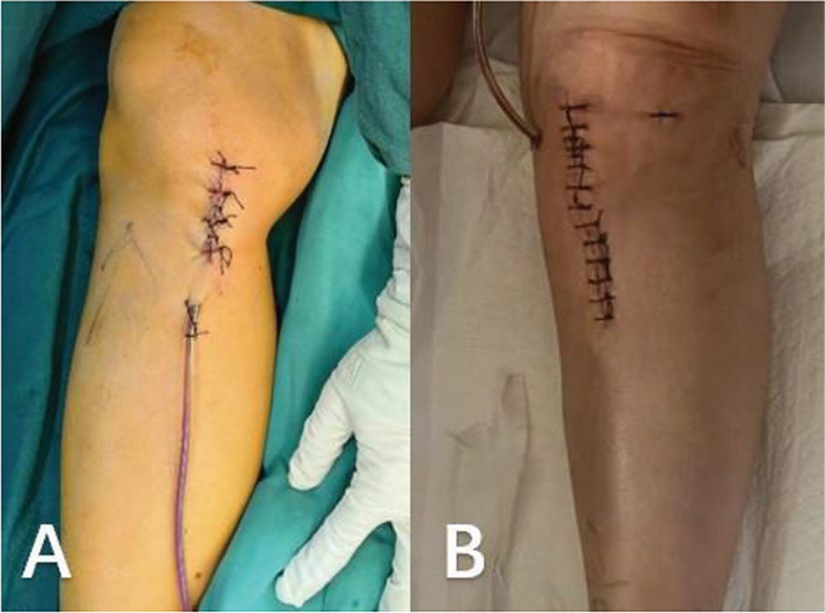
**A**, group1(DT-LCP) incision photograph; **B**, group2(DT-LCP) incision photograph

The surgical procedures were performed under general anesthesia. The patient was placed in the supine position on the operating table. A tourniquet was used during the operation. A longitudinal incision of approximately 5 cm in length was made medial to the knee joint, and the skin, subcutaneous tissue, and deep fascia were incised sequentially to expose the pes anserinus and medial collateral ligament. Then, under fluoroscopy, a guide needle was inserted at the edge of the pes anserinus toward the fibular head, and then a 2.0 mm Kirschner wire was used to cut the bone through the guide (Fig. 3). The bone chisel (Fig. 4) was used for osteotomy, and according to the preoperative plan, the test mold (Fig. 5) was used to open the osteotomy space from small to large until the target angle was reached, and fixed with DT-LCP (Figs. 6 and 7: A, B). Finally, filler was inserted into the osteotomy gap (51patients used the autogenous bone, 24 patients used allograft bone implant material, 19 patients used the absorbable spacer and 4 patients did not fill the gap) and the wound was rinsed and sutured (Fig. 8: A, B).

The osteotomy uses Kirschner wire and bone chisel, which locates the osteotomy more accurately and solves the defect that the oscillating saw easily caused nerve, blood vessel, and soft tissue damage. In DT-LCP group, we do not think it is necessary to dissect the posterior periosteum and insert a retractor to protect the neurovascular tissues prior to the osteotomy as we did in the T-LCP group. The bone chisel is much less destructive than the oscillating saw, and the cutting range is easier to control. The test mold is divided into different types and the upper part is thin and the lower part is thick. We can use different test molds to adjust the varus angle and posterior tibial slope.

### Group2 (T-LCP)

The surgical procedures were performed under general anesthesia. The patient was placed in the supine position on the operating table. A tourniquet was used during the operation. A longitudinal incision of approximately 8 cm in length was made at 1.5 cm medial to the tibial tuberosity. The skin and subcutaneous tissue were incised sequentially to expose the pes anserinus. The soft tissue was separated under the periosteum to reveal the osteotomy position. Two Kirschner wires were inserted obliquely into the upper edge of the pes anserinus for positioning, and the C-arm fluoroscopy was well positioned for osteotomy using a oscillating saw. The medial tibia was propped open, the mechanical axis of the lower limb was measured, the T-LCP was placed (Fig. 7: C, D), the holes were drilled in sequence to measure the depth, the screws were placed, and the C-arm fluoroscopic plate screws were positioned at the appropriate length. Finally, filler was inserted into the osteotomy gap (99 patients used the allograft bone implant material implant material, and 5 patients did not fill the gap) and the wound was rinsed and sutured (Fig. 8: C, D).

### Rehabilitation

After the operation, patients were encouraged to exercise knee joint function in bed to prevent joint stiffness, and the range of motion was not limited. Partial weight-bearing exercise was performed with the assistance of a walker 2 to 3 d after surgery, and weight-bearing functional exercise was gradually started 2 months after surgery when the bone at the osteotomy healed.

### Evaluation

Patients were screened for baseline characteristics such as gender, height and weight, age, body mass index (BMI), the severity of osteoarthritis (Kellgren-Lawrence classification, range 0–4), and length of surgical incision. Clinical evaluation scores were collected before the operation and at last follow-up. Clinical evaluation included the International knee documentation committee knee evaluation form (IKDC) [18], Knee Injury and Osteoarthritis Outcome Score (KOOS) [19] and Western Ontario and McMaster Universities Osteoarthritis Index (WOMAC) [20].

To determine whether the improvement in outcomes was clinically meaningful, the minimum clinically significant difference (MCID) for each assessment scale was used as a reference value [21, 22], which were reported in osteoarthritis literature. We had compared the proportion of patients whose clinical outcomes improved beyond the minimum clinically important difference (MCID) between the groups.

### Statistical analysis

Statistical analysis was carried out using IBM SPSS statistics version 20.0 software (IBM Inc., Chicago, IL). Categorical variables were expressed as numbers and percentages (%), and continuous variables were expressed as mean ± standard deviation (SD) and range. If the continuous variable obeyed normal distribution, independent sample t-test was used; otherwise, Mann-Whitney tests were used. The categorical variables were determined by the Chi-square test or Fisher’s test. The proportion of patients whose clinical score was higher than that of MCID was compared between groups by Fisher’s test or Chi-square test. *P* < 0.05 was considered significant.

## Results

After applying the exclusion criteria, 202 patients with KOA (202 knees) were included in the analysis, including 143 females and 59 males, aged from 39 to 81 years (64 ± 7). The average follow-up time was 21 ± 8 months. According to the data, group 1 (DT-LCP) patients were 98 (48.5%), and group 2 (T-LCP) patients were 104 (51.5%). Table 1 summarizes the baseline characteristics. Both groups were similar in terms of age, follow-up time, gender, BMI, tobacco, ASA score, Kellgren-Lawrence grade and angle for correction. Age and BMI were slightly higher in group 1 (DT-LCP) than in group2 (T-LCP). The incision length of group 1 (DT-LCP) is significantly smaller than that of group 2 (T-LCP).

**Table 1:** Comparison of patient demographics by group

		Group 1(*n* = 98)^a^			Group 2 (*n* = 104)^a^		*P* value
Age at surgery(year)		64.2 ± 8.5 [43,81]			63.0 ± 5.0 [39,78]		**0.006** ^*^
Follow-up (months)		21.0 ± 9.0 [12,52]			20.4 ± 5.8 [12,38]		0.620
Sex (female)		70 (71.4%)			73(70.2%)		0.847
BMI		27.1 ± 2.8 (21, 34)			26.2 ± 2.8 [19,39]		**0.022** ^*^
Tobacco ASA score		3 (5%)			3 (7.1%)		1.000
ASA 1		7 (7.1%)			5(4.8%)		0.483
ASA 2		68 (69.4%)			75 (72.1%)		0.670
ASA 3		23 (23.5%)			24 (23.1%)		0.947
Kallgren-Lawrence grade							
2		20(20.4%)			25 (24.1%)		0.535
3		61 (62.2%)			67 (64.4%)		0.748
4		17 (17.3%)			12 (11.5%)		0.239
Incision Length		5.0 ± 0.4 (4, 6)			8.0 ± 1.2 (5, 12)		**< 0.001** ^*^
Preoperative mFTA (°)		173.0 ± 1.9 [166,178]			173.1 ± 1.9 [167,178]		0.600
Postoperative mFTA (°)		180.9 ± 1.8 [174,185]			180.8 ± 1.6 [175,184]		0.630
Preoperative IKDC^**b**^		42.2 ± 13.6 [20,69]			44.5 ± 12.7 [20,69]		0.258
Preoperative KOOS^**c**^							
Pain		48.8 ± 8.1 [33,64]			50.5 ± 11.2 [31,72]		0.306
Symptoms + stiffness		50.3 ± 10.5 [29,64]			51.2 ± 12.0 [29,75]		0.521
Daily living		57.4 ± 7.2 [43,69]			58.2 ± 8.6 [43,79]		0.572
Sports		28.3 ± 9.4 [5,50]			29.2 ± 10.5 [5,45]		0.519
Quality of life		27.9 ± 14.1 [6,56]			30.0 ± 16.6 [6,63]		0.481
Preoperative WOMAC^**d**^							
Pain		63.8 ± 9.6 [45,80]			62.7 ± 10.7 [35,75]		0.323
Stiffness		49.1 ± 10.5 [13,63]			47.5 ± 12.8 [13,63]		0.555
Function		59.4 ± 6.1 [47,68]			58.3 ± 7.5 [35,68]		0.625
Total		59.5 ± 6.6 [47,69]			58.3 ± 7.8 [39,69]		0.415

BMI body mass index (kg/m^2^); ASA American Society of Anesthesiologists; mFTA, mechanical femoro-tibial angle; IKDC, The International knee documentation committee knee evaluation form; KOOS, Knee Injury and Osteoarthritis Outcome Score; WOMAC, Western Ontario and McMaster Universities Osteoarthritis Index. ^**a**^Data are presented as mean ± standard deviation [minimum, maximum] or number (proportion). ^**b**^Score range, 0 to 100; 0 indicates extreme symptoms. ^**c**^Score range, 0 to 100; 0 indicates extreme symptoms; Score range, 0 to 100; 0 indicates no symptoms. ^**d**^Score range, 0 to 100; 0 indicates no pain. * Represents that *P* < 0.05

### Clinical outcomes

Detailed clinical results are summarized in Table 2. The KOOS pain score and the WOMAC pain score increased in both groups from preoperative to the final follow-up. We found that the difference between the KOOS pain score (*P* = 0.040) and the WOMAC pain score (*P* = 0.023) at the final follow-up was statistically significant and the results were in favor of group1(DT-LCP). In the subscale, group 1(DT-LCP) had better daily living score (*P* = 0.032, KOOS), function score (*P* = 0.048, WOMAC) than group 2(T-LCP). Regarding the improvements between preoperative and the last follow-up of pain score (*P* = 0.006, KOOS), daily living score (*P* = 0.008, KOOS), pain score (*P* = 0.023, WOMAC), function score (*P* = 0.019, WOMAC) and total score (*P* = 0.009, WOMAC) were in favor of group 1. In IKDC score, group 1 (DT-LCP) was significantly better than group 2 (T-LCP) (*p* < 0.001). KOOS and WOMAC improved over MCID at the last follow-up in both groups compared to preoperative, but there was no significant group difference between the two groups (Table 3).

**Table 2:** Clinical outcomes for each group

Variable	Group 1(*n* = 98)^a^	Group 2 (*n* = 104)^a^	*P* value	
Postoperative IKDC (at one year follow-up)	81.5 ± 15.1 [34,97]	75.2 ± 19.5 [21,97]		**0.025** ^*^
IKDC improvement	37.5 ± 10.9 [15,56]	30.7 ± 14.0 [-12,53]		**< 0.001** ^*^
Postoperative KOOS (at one year follow-up)				
Pain	85.9 ± 12.7 [42,100]	83.8 ± 12.9 [42,97]		**0.040** ^*^
Symptoms + stiffness	84.8 ± 13.8 [43,100]	83.3 ± 13.9 [43,100]		0.356
Daily living	86.8 ± 11.5 [56,100]	83.9 ± 11.8 [44,99]		**0.032** ^*^
Sports Quality of life	57.2 ± 14.0 [20,100] 75.4 ± 20.0 [19,100]	56.1 ± 13.8 [10,100] 73.7 ± 20.0 [19,100]		0.657 0.497
KOOS improvement				
Pain	37.1 ± 12.8 [0,61]	33.4 ± 12.6 [-6,61]		**0.006** ^*^
Symptoms + stiffness	34.5 ± 17.2 [-18,68]	32.1 ± 16.8 [-18,61]		0.257
Daily living	29.3 ± 11.0 [-7,53]	25.7 ± 11.0 [-12,49]		**0.008** ^*^
Sports Quality of life	28.9 ± 15.0 [-15,80] 47.6 ± 23.8 [-19,88]	26.9 ± 13.7 [-10,55] 43.8 ± 23.8 [-31,88]		0.334 0.199
Postoperative WOMAC (at one year follow-up)				
Pain	18.9 ± 16.5 [0,65]	21.0 ± 14.2 [0,70]		**0.023** ^*^
Stiffness	21.0 ± 11.9 [0,63]	23.1 ± 14.4 [0,63]		0.581
Function	22.8 ± 14.9 [0,59]	27.1 ± 14.7[0,68]		**0.048** ^*^
Total	22.0 ± 14.2 [0,60]	25.5 ± 14.0 [0,68]		0.054
WOMAC improvement				
Pain	44.9 ± 16.4 [-10,70]	41.7 ± 14.7 [-15,65]		**0.023** ^*^
Stiffness	28.1 ± 14.4 [-13,50]	24.4 ± 14.7 [-25,50]		0.081
Function	36.6 ± 15.5 [-1,65]	31.2 ± 15.1 [-15,53]		**0.019** ^*^
Total	37.6 ± 14.4 [-4,61]	32.9 ± 13.7 [-14,51]		**0.009** ^*^

IKDC, The International knee documentation committee knee evaluation form; KOOS, Knee Injury and Osteoarthritis Outcome Score; WOMAC, Western Ontario and McMaster Universities Osteoarthritis Index. ^**a**^Data are presented as mean ± standard deviation [minimum, maximum] or number (proportion); * Represents that *P* < 0.05

**Table 3:** The minimal clinically important difference (MCID) between two groups

	MCID^a^	Group 1(*n* = 98)	Group 2(*n* = 104)		*P* value
KOOS					
Pain	15.4	91 (92.9%)	97 (93.3%)		0.908
Symptoms + stiffness	15.1	88 (89.8%)	96 (92.3%)		0.531
Daily living	17.0	88 (89.8%)	93 (89.4%)		0.931
Sports	11.2	89 (90.8%)	93 (89.4%)		0.911
Quality of life	16.5	90 (91.8%)	95 (91.3%)		0.900
WOMAC					
Pain	11.0	91(92.9%)	97(93.3%)		0.908
Stiffness	8.0	92(93.9%)	94(90.4%)		0.358
Function	9.0	91(92.9%)	95(91.3%)		0.691
Total	10.0	90(91.8%)	97(93.3%)		0.698

^**a**^The minimal clinically important difference; KOOS, Knee Injury and Osteoarthritis Outcome Score; WOMAC, Western Ontario and McMaster Universities Osteoarthritis Index

### Complications

A total of 54 patients (26.7%) experienced complications in our study and there was a significant difference between the two groups(*P* = 0.022). In group 1 (DT-LCP), there were 19 complications (19.4%) (2 implant irritation requiring plate removal, 10 deep vein thrombosis, 3 weakness of the leg, 3 infections, and 1 lateral hinge fracture). In group 2 (T-LCP), there were 35 complications (33.7%) (15 implant irritation requiring plate removal, 11 deep vein thrombosis, 4 weakness of the leg, 3 infections, and 2 lateral hinge fractures). There was a significant difference in implant irritation requiring plate removal between the two groups (*P* = 0.004), and the result was beneficial to group 1 (DT-LCP).

There was no bone nonunion and implant failure in both groups. During the follow-up period, no patients were found to have been converted to total knee arthroplasty (TKA). Details of the complications are recorded in Table 4.

**Table 4:** Comparison of complication rates between two groups

	Group 1 (*n* = 98)^a^	Group 2 (*n* = 104)^a^	*P* value		
Complications from the bone Non-union requiring revision	0 (0%)	0 (0%)	NS		
Lateral hinge fractures	1 (1.0%)	2 (1.9%)	NS		
Complications from the implant					
Implant irritation requiring plate removal	2(2%)	15(14.4%)	0.004		
Implant failure Generalized complications	0 (0%)	0(0%)	NS		
All Infections	3 (3.1%)	4 (3.8%)	NS		
DVT	10(10.2%)	11(10.6%)	0.931		
Weakness of the leg	3(3.1%)	3 (2.7%)	NS		
Total	19(19.4%)	35 (33.7%)	0.022*		

^**a**^Data are presented as number (proportion); DVT, deep vein thrombosis; * Represents that *P* < 0.05; NS, not statistically significant

## Discussion

The most important findings of our study are that the mean pain scores (KOOS, WOMAC) at the final follow-up were significantly higher among group 1 compared to group 2 (*P* = 0.040 and *P* = 0.023). Furthermore, the DT-LCP internal fixation exerted more excellent effects on other symptoms, function and quality of life than T-LCP internal fixation. No severe adverse effects occurred for both treatments, but the incidence of complications in group1 is lower than that in group2 (*P* = 0.022).

During the last 15 to 20 years, the KOOS scores and the WOMAC scores have been proved to accurately reflect patients’ views on knee joint function and can be used as effective tools to evaluate the treatment outcome of knee osteoarthritis [23–25]. Over the past decades, TomoFix™ (Synthes, Switzerland) is widely used in high tibial osteotomies because of its superior clinical results [14, 26], and more internal fixation plates of similar construction have emerged [27]. The T-LCP used in the control group of this study had a similar structural size to TomoFix™. Although the incidence of complications with TomoFix™ is low, its large size may lead to discomfort in the early rehabilitation stage [28, 29]. A possible explanation for implant irritation requiring plate removal relates to the limited soft tissue in the anterior medial aspect of the tibia [15]. In previous studies, it has been demonstrated that lower implants can reduce implant irritation and promote patient recovery [30, 31]. However, there is no internal fixator with a corresponding effect in high tibial osteotomy. Hi-Un Park et al. [32]. reported, for instance, that they used a smaller plate(LOQTEQ^®^ HTO Plate, absolute plate length is 90 mm.) for internal fixation, but the clinical results were not statistically different from those of TomoFix™. Therefore, the new implant designed in this study takes a smaller size and more stable double triangular support structure, which is 60 mm long and has a smaller volume. The scores’ improvements in patients observed in this study highlight the benefits of our design changes. Furthermore, in terms of implant irritation, DT-LCP has a lower incidence (*P* = 0.004). Implant irritation causes the patient pain requiring a second surgery to remove the internal fixation. The rate of internal fixation removal in other studies was about 7% [4], whereas in the present study, the incidence was only 2%. It can be concluded that DT-LCP can reduce implant irritation and thus relieve patient pain. Our study demonstrates that when the volume of the internal fixation plate is reduced to the size we designed, it can effectively reduce pain and thus promote early recovery of the patient.

In this study, patients with DT-LCP internal fixation achieved better knee function compared to T-LCP. Satisfactory functional outcomes are associated with minimally invasive approaches. All patients in the first group underwent internal fixation with DT-LCP, a technique that resulted in a lower incidence of soft tissue problems and complications and achieved better outcomes than T-LCP. In addition. The surgical incision was approximately 5 cm for group 1 (DT-LCP) and 8 cm for Group 2 (T-LCP). The former had a significantly smaller incision (*P* < 0.001). Using DT-LCP will lead to smaller incisions and less damage to the surrounding soft tissues for patients, which can reduce postoperative pain and enable early mobility, thereby improving joint nutrition and promoting fracture healing. Due to less scar formation, the joint function is better. As shown in Table 2 of our study, the KOOS score and WOMAC score also confirmed this. DT-LCP internal fixation is more friendly to patients who care about the size of the incision. In our study, we found that DT-LCP effectively reduced the rate of secondary surgery (from 15 to 2%) compared to T-LCP. This reduces the harm of having a second surgery as well as lowering the cost of treatment for the patient.

In terms of adverse events, the most common complication not associated with the implant was deep vein thrombosis (DVT), which required treatment with low molecular weight heparin. Another study showed that early DVT after OWHTO did not cause adverse outcomes [33]. The results of our study confirm this point. We advocate routine thromboprophylaxis after high tibial osteotomy, especially in high-risk groups. Although early ambulation and functional exercise can prevent venous thrombosis of the lower extremities, some patients are not able to strictly follow the doctor’s instructions because of the pain after osteotomy. In other studies, the incidence of DVT after high tibial osteotomy was 2.4–44.7% [34]. The incidence of deep venous thrombosis in both groups was 10%. Active thrombus prevention is helpful in preventing the occurrence of DVT.

Although the early clinical outcome of DT-LCP is better, the long-term stability of DT-LCP is unknown. DT-LCP has only 6 locking screws, and the holding power is poorer than T-LCP with 8 locking screws. DT-LCP is short and stress is concentrated, which may affect the long-term stability, but it is not found in the follow-up and needs long-term observation.

### Limitations

The data in this study are from a single medical center and further studies are needed based on other medical centers to test the universality of our results. This is a retrospective study and the data collection process may affect the authenticity and accuracy of the information. The rating scales were used to assess the surgical outcome, and the patient’s physical and psychological status would affect the authenticity and accuracy of filling out the rating scale. This study included 202 patients, the sample size is small, and there may be false negatives. The follow-up time was short, the average follow-up time was 21 months, and the long-term effect of the operation remained to be observed. We have not evaluated the effect of articular cartilage changes and should use ultrasound or magnetic resonance imaging for further studies.

## Conclusions

A comparison of two different plates (DT-LCP vs. T-LCP) following OWHTO showed that DT-LCP has better clinical outcomes and lower complication rates. It can, currently, be concluded that the DT-LCP is a reliable and safe implant for OW-HTO, and long-term effects and safety need to be further observed. It can be used as an alternative for patients who are concerned about the length of the incision.

## Data Availability

No datasets were generated or analysed during the current study.
